# Antibiotic-Powered Energy Harvesting: Introducing Benzylpenicillin as an Efficient Tribopositive Material for Triboelectric Nanogenerators

**DOI:** 10.3390/nano13232995

**Published:** 2023-11-22

**Authors:** Asad Nauman, Shahid Ameen, Hak-Rin Kim

**Affiliations:** 1 School of Electronic and Electrical Engineering, Kyungpook National University, Daegu 41566, Republic of Korea; naumanasadknu@knu.ac.kr; 2 Department of Chemistry, Ulsan National Institute of Science and Technology (UNIST), Ulsan 44919, Republic of Korea; sasamra313@unist.ac.kr; 3 School of Electronics Engineering, Kyungpook National University, Daegu 41566, Republic of Korea

**Keywords:** benzylpenicillin sodium salt, energy harvesting, triboelectric nanogenerator, biomaterial, antibiotics, sustainability

## Abstract

The pursuit of enhancing the performance of triboelectric nanogenerators (TENGs) has led to the exploration of new materials with efficient charge-generating capabilities. Herein, we propose benzylpenicillin sodium salt (b-PEN) as a candidate biomaterial for the tribopositive layer owing to its superior electron-donating capability via the lone pairs of electrons on its sulfur atom, carbonyl, and amino functional groups. The proposed b-PEN TENG device exhibits promising electrical performance with an open-circuit voltage of 185 V, a short-circuit current of 4.52 µA, and a maximum power density of 72 µW/cm^2^ under force applied by a pneumatic air cylinder at 5 Hz. The biomechanical energy-harvesting capabilities of the b-PEN TENG device are demonstrated by actuating it with finger, hand, and foot movements. Moreover, the proposed TENG device is utilized to charge capacitors and power light-emitting diodes by scavenging the externally applied mechanical energy. This outstanding electrical performance makes b-PEN a promising tribopositive material.

## 1. Introduction

Novel wireless and wearable electronic devices in the fields of healthcare monitoring, environmental sensing, and the Internet of Things require portable electrical energy sources for continuous operation [1,2,3,4]. To overcome the power limitations of these applications, portable battery–operated electronic devices are expected to be replaced with flexible low-power wearable devices in the upcoming decades [5,6]. Furthermore, the emergence of self-powered energy sources that harness energy from their environment using nanomaterials and nanotechnologies, is a rapidly growing domain of nanoenergy electronics [2,7,8,9]. Particularly, mechanical energy generated through body movement can be harvested to power wearable electronics, thereby avoiding energy waste [10,11,12,13]. To date, several types of nanogenerators, including triboelectric [14], piezoelectric [15,16], electromagnetic [17], and thermoelectric [18,19], have been designed to transform the mechanical and thermal energies of the human body into electrical energy.

Triboelectric nanogenerators (TENGs) have been demonstrated to generate power by converting mechanical energy into electrical energy [20]. This conversion of mechanical energy into electrical energy involves the coupling effect of contact triboelectrification and electrostatic induction. Fundamentally, TENGs operate based on Maxwell’s displacement current and change in the surface polarization of the material [14]. Over recent years, TENGs have attracted the attention of researchers owing to their remarkable energy conversion efficiency, promising output capabilities, cost-effectiveness, and ease of fabrication [21,22]. Plenty of TENG-powered systems have demonstrated the potential to effectively harvest renewable energy from ocean waves [23], vibrations [24], wind [25], and rain [26], assisting the existing energy technologies in energy generation. Various approaches, such as the selection of appropriate materials, optimization of the device structure, and power management circuit methods have been utilized to improve the electrical performance of TENGs [21,27]. Specifically, the selection of tribomaterials and nanoscale modifications are crucial for ensuring the high electrical performance of TENG devices. Polytetrafluoroethylene (PTFE) is widely adopted as an efficient tribonegative layer for TENG devices because of its high electron affinity [21,28]. At the same time, the selection of positive triboelectric materials is more limited than that of tribonegative materials [29]. The output electrical energy of the TENG device is quadratically proportional to the surface charge density (σ_sc_) [21,27]; therefore, more efficient tribopositive materials must be developed to considerably improve TENG performance. The widely adapted tribopositive materials that tend to easily lose electrons include polyamide (PA), polypyrrole (PPy), and polyethylene (PE) [30,31,32]. Despite the promising energy harvesting performance of synthetic polymers, in the past decades, the renewable and sustainable energy sources that can have a positive impact on the environment, have gained attention. Recently, researchers have focused on the recycling of abundant waste materials such as plastic [33], aluminum soda can trash [34], rubber [35], and waste papers [36] for TENGs as sustainable clean energy. However, these waste materials in their raw state are highly uncomfortable and therefore require careful handling and processing for their use in energy harvesting devices [37]. In the past decade, researchers have explored a range of biomaterials for wearable devices, such as lignin [38,39], cellulose [30,31,40,41], tomato peels [42], silk [43,44], peanut [45], and sunflower outer shells [46], which have shown promising tribopositive properties.

Penicillin G, generally known as benzylpenicillin, is of high significance in the field of antibiotics and serves as a fundamental compound for the production of semisynthetic penicillin [47,48]. Globally, more than 20 nations produce penicillin, totaling more than 11,000 metric tons annually [48]. Benzylpenicillin sodium salt (b-PEN) can be considered a promising candidate for triboelectric nanogenerators because of its compatibility, lightweight, and low cost. The electrical properties of b-PEN can be attributed to the presence of an amide group (–NH–CO–) in the lactam ring. In the chemical transition stage of b-PEN from its neutral molecular form to its anion form (as in its sodium salt), the dipole moment increased from 4.7 Debye to 18.2 Debye [49]. This specific chemical structure of b-PEN provides a specific surface charge distribution over the molecules, which could help enhance the electrical performance of the triboelectric nanogenerators when employed as tribopositive material.

Herein, we propose benzylpenicillin sodium salt (b-PEN) for use in TENG devices as an energy-harvesting biomaterial. We demonstrate that b-PEN is a tribopositive material owing to its excellent electron-donating capability, which is attributed to the existence of lone electron pairs on its sulfur atom, carbonyl, and amide functional groups (–NH–CO–) in the lactam ring [50,51]. The proposed TENG device was assembled using the b-PEN as the tribopositive layer and PTFE as the tribonegative layer. The fabricated device exhibited excellent electrical performance (185 V, 4.52 µA, and 72 µW/cm^2^) at a frequency of 5 Hz. The electrical output of the device was evaluated at lower frequencies of 0.5, 1, and 3 Hz, exhibiting promising performance for low-frequency energy harvesting applications. Various performance tests were performed to ensure the stability and reliability of the fabricated device. Moreover, the performance of the device was demonstrated when using actuation through body movements. Finally, the practical application of the b-PEN TENG device as a self-powering energy source was assessed by driving several light-emitting diodes (LEDs) and charging capacitors.

## 2. Materials and Methods

### 2.1. Uniform Coating of b-PEN through Roll Pressing

**Figure 1:**
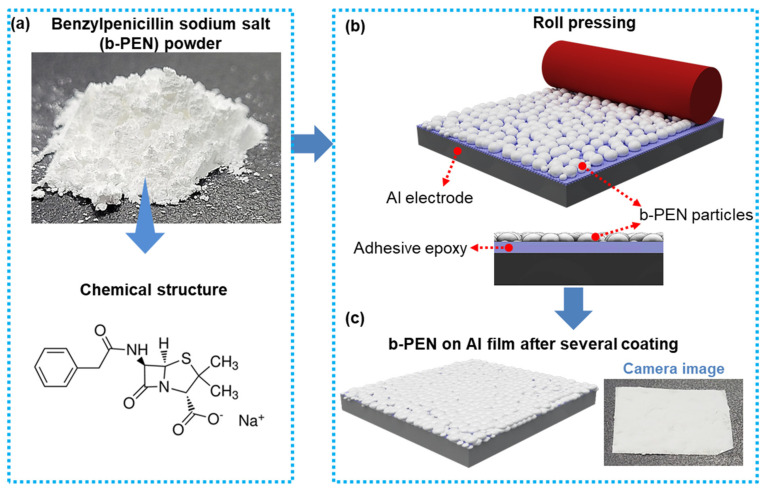
(**a**) Benzylpenicillin sodium salt (b-PEN) powder and its chemical structure. (**b**) Process of uniformly coating the surface of the Al electrode with b-PEN. (**c**) Schematic and camera image of b-PEN coated Al tape as a tribopositive layer.

A photo of the as-purchased b-PEN (Sigma Aldrich, Seoul, Republic of Korea) in powder form is shown in Figure 1a. The chemical structure of b-PEN consists of a beta-lactam ring fused with a thiazolidine ring. Electron-donating and electron-accepting natures of the electropositive and electronegative materials, respectively, play a critical role in producing triboelectric charge; therefore, choosing a triboelectric material is a top priority in designing TENGs. In b-PEN, a benzyl group is attached to the beta-lactam ring through an amide group. The amide functional group is characterized by a carbonyl group (C=O) bonded to a nitrogen atom. Two amide groups are present in b-PEN, one in the lactam ring and the other outside the ring to which the benzyl group is attached. The amine (cyclic amide in lactam ring) along with the carbonyl group (–C=O) present in penicillin’s structure, induce a specific charge distribution over the molecule [52]. This results in an interfacial dipole with a magnitude of 4.7 Debye [49]. Thio (S), and amide (–NH–CO–) groups in the lactam ring are electron-rich, so they could easily donate electrons, hence making b-PEN a potential tribopositive material [52,53].

A schematic of the procedure employed to uniformly and firmly coat b-PEN on the adhesive surface of the Al film is shown in Figure 1b. The b-PEN powder was uniformly distributed on the surface of the Al film and repeatedly roll-pressed using a hand-held rolling machine. Rolling and pressing ensures the complete blending of b-PEN powder with the acrylic adhesive on the Al tape. The acrylic adhesive is thermally stable, moisture-stable, chemically stable, and highly adhesive compared to rubber adhesives [54,55]. These properties enable the uniform and stable coating of the b-PEN powder on the Al tape. The process was repeated several times to completely fill the surface of the Al film to obtain a stable and uniformly thin layer of tribopositive b-PEN on the surface of Al. Figure 1c shows the schematic and camera image of Al tape coated with b-PEN powder.

### 2.2. Characterization

**Figure 2:**
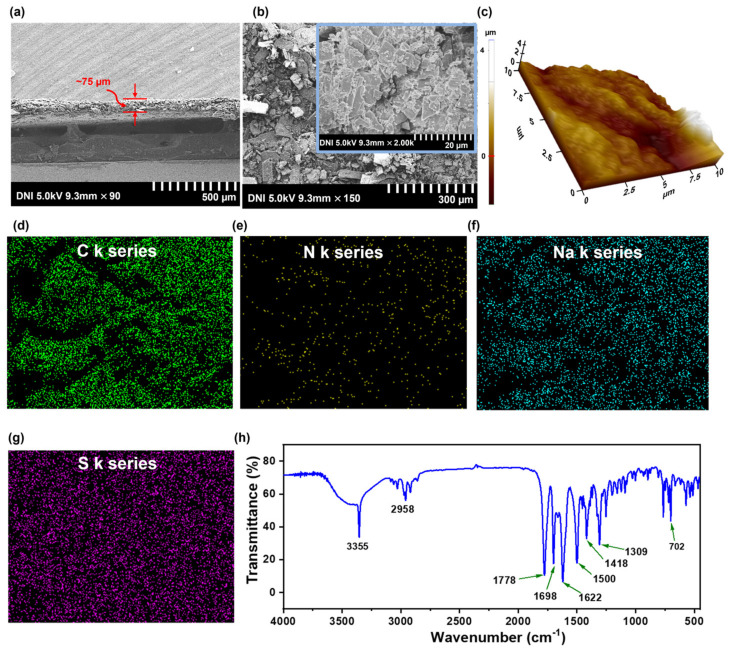
(**a**) Field−emission scanning electron microscopy (FE−SEM) image of the cross−section of b-PEN tribopositive layer coated on the Al film. (**b**) Top-view FE−SEM image of b-PEN (scale bar: 300 µm) with inset image at higher magnification (scale bar: 20 µm). (**c**) 3D AFM nanaoprofile image of the bPEN film. (**d**–**g**) Energy−dispersive spectroscopy results for the b-PEN, confirming the presence of C k, N k, Na k, and S k series. (**h**) Fourier transforms infrared spectrum of b-PEN.

The surface morphology and the elemental maps of the uniformly coated b-PEN tribopositive film were investigated using field-emission scanning electron microscopy (FE-SEM) with energy-dispersive spectroscopy (EDS) Oxford detector, as shown in Figure 2. The cross-sectional FE-SEM image of the b-PEN film is shown in Figure 2a, where tribopositive film has a thickness of ~75 μm. The surface morphology of the tribopositive layer significantly contributes to the device’s performance by enhancing the surface contact area and consequently maximizing the surface charge density. Figure 2b exhibits the top-view FE-SEM image showing the pellet-shaped structure with round particles at the magnification of 20 μm. The 3D nanoprofile of b-PEN coated on Al tape measured with atomic force microscopy (AFM) is shown in Figure 2c. The surface roughness was estimated to be 2.1 µm. The surface profile shows high roughness with minimal discontinuity which could help achieve higher output performance. The elemental analysis of the b-PEN film was performed through EDS, with the results (Figure 2d–g) confirming the presence of C k, N k, Na k, and S k series. The high concentrations of electron-donating elements such as S and N indicate the tribopositive properties of b-PEN. Figure 2h shows the Fourier transform infrared spectroscopy (FT-IR; IR200 Thermo Fisher Scientific, Waltham, MA, USA) results for the b-PEN thin film. The band observed at 3355 cm^−1^ is associated with the amide N-H stretching. 1778 cm^−1^ is associated with the stretching of the CO bond of the β−lactam The peaks at 1698 and 1622 cm^−1^ are associated with amide and asymmetric stretching of the carboxylate, respectively. The band observed at 1309 cm^−1^ represents the secondary amide group and the one at 702 cm^−1^ represents N-H deformation in secondary amides.

### 2.3. Device Fabrication

**Figure 3:**
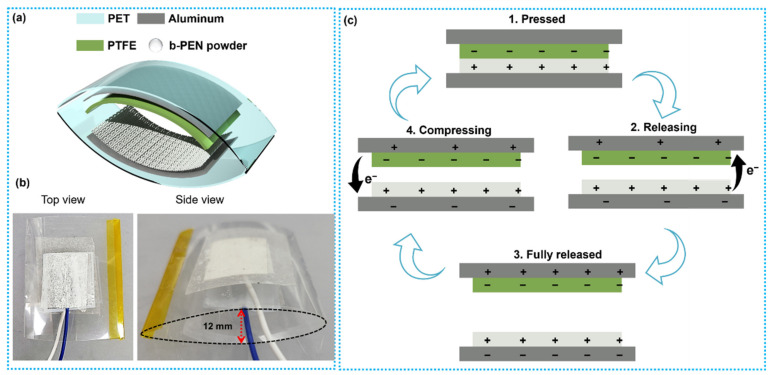
(**a**) Schematic of the fabricated b-PEN-based triboelectric nanogenerator (TENG) device, (**b**) photos of the fabricated TENG device showing the top and side view, and (**c**) electron flow mechanism in the fabricated TENG during mechanical deformation.

The proposed TENG device was fabricated using b-PEN and PTFE (Sigma Aldrich, Seoul, Republic of Korea) as triboelectric layers in contact–separation mode. The schematic and photo of the fabricated arc-shaped TENG device are shown in Figure 3a,b. In the proposed TENG device, b-PEN acts as a tribopositive layer, while PTFE acts as a tribonegative material. The generated electric potential is collected using an adhesive Al tape as a flexible electrode. A flexible sheet (PET) with an arc-shaped design was used as a supporting substrate for the electrodes. This substrate provided sufficient support for the quick self-recovery of the device to a fully released state in the contact–separation process. The b-PEN powder was uniformly spread on the adhesive surface of the Al tape and pressed to ensure adhesion. At the same time, PTFE was attached to the other Al electrode (adhesive side). Copper wires for external electrical connection were attached to the nonadhesive sides of the Al electrodes. The active surface area of the fabricated TENG device was 2 × 2 cm^2^, whereas the distance between the two triboelectric layers was ~12 mm at the curvature center of the arc-shaped TENG in the original state, as shown in the side view of Figure 3b.

### 2.4. Electrical Measurement

**Figure 4:**
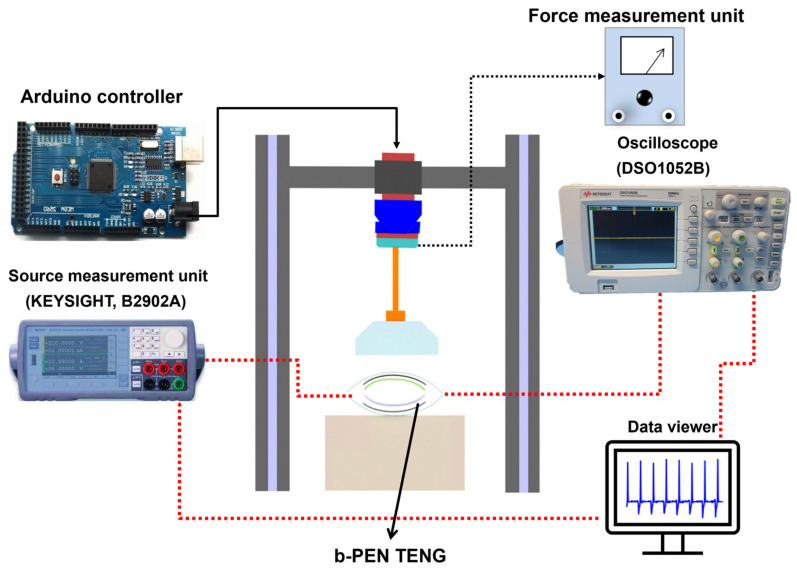
Schematic illustration of the dynamic testing platform used to measure the electrical output performance of the b-PEN under different frequencies.

We have measured the electrical performance of the b-PEN TENG device using a standard dynamic push testing benchtop platform as illustrated in Figure 4. The pushing tester device can be operated under different frequencies and forces to test the electrical output of TENGs in contact separation mode. An Arduino controller was utilized to program the operating frequency and normal force applied by the pushing cylinder. The cyclic uniform force was applied to the TENG device using a controllable pneumatic air cylinder (Shinyeong Mechatronics Co. Ltd., C3B16-50, Siheung-si, Gyeonggi-do, Republic of Korea). A force sensor that is connected to the pushing tester and accurately measures the applied pressing force. The acrylic block attached to the cylindrical shaft at the bottom contacts the b-PEN TENG to generate the output signal. Open-circuit voltage was measured using an oscilloscope (Agilent Tech., DSO1052B, Santa Rosa, CA, USA), whereas the current of the b-PEN/PTFE TENG device was obtained using a source measurement unit (KEYSIGHT, B2902A, Santa Rosa, CA, USA). The data obtained from the measurement devices was then plotted using Origin Pro 8.5.1 (Origin Lab Corporation, Northampton, MA, USA).

## 3. Results and Discussion

The proposed TENG device operates through the contact–separation mechanism, as depicted in Figure 3c. When the external force is applied to the TENG device, the top tribopositive layer and bottom tribonegative layer contact each other, as shown in Figure 3c (1). As a result, equal negative and positive charges are generated on the tribonegative and tribopositive sides. When the external force is removed, induced charges accumulate on the top and bottom electrodes, opposite to the dielectric layers, during the release, as shown in Figure 3c (2). This accumulation of separated charges is caused by electrostatic induction. Subsequently, the charges flow into the external circuitry attached to the electrodes. After the triboelectric layers are released and completely separated, a charge equilibrium is attained between the top and bottom layers, and the current momentarily disappears in this equilibrium state, as shown in Figure 3c (3). When both layers are brought closer to each other again by the external force, the electric potential starts rebuilding, and the current starts flowing in the opposite direction, as shown in Figure 3c (4). As a result, an alternating current is generated under cyclic application of force to the fabricated TENG device.

**Figure 5:**
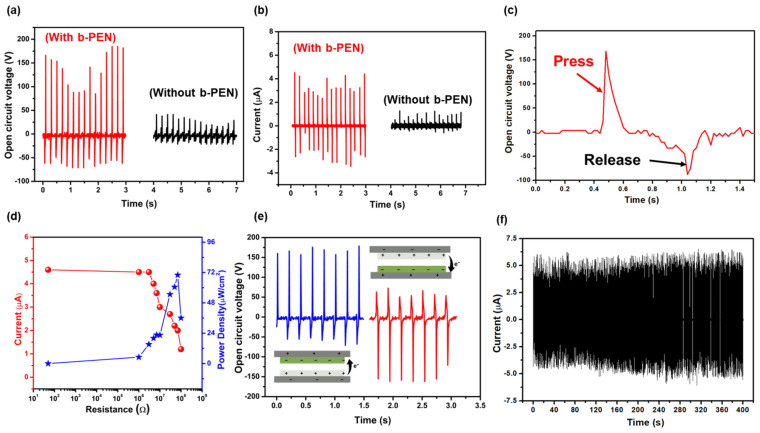
(**a**) The open−circuit voltage of the proposed TENG device under repeated mechanical force with and without b-PEN (tribopositive layer). (**b**) Short−circuit current of the proposed TENG with and without b-PEN layer. (**c**) Zoomed view of the output voltage signal in a single pressing and releasing cycle. (**d**) Maximum instantaneous power density against resistive load, where the red line with dots indicates the current and the blue line with stars indicates the power density. (**e**) Phase−change analysis of the proposed b-PEN−based TENG device, the blue line indicates a forward connection while the red line indicates a reverse connection when measuring the voltage signals between the electrodes. (**f**) The stability test of the b-PEN-based TENG under repeated excitation for 2000 cycles.

Figure 5 exhibits the electrical performance of the fabricated b-PEN/PTFE-based TENG device under an excitation force of 20 N at 5 Hz frequency under ambient environmental conditions. Factors important for sustainable energy harvesting include voltage, current, and instantaneous power of the device. The proposed b-PEN/PTFE-based TENG devices generate an output voltage of ~185 V and a maximum short-circuit current of ~4.52 µA (Figure 5a,b). The zoomed view of the voltage signal in a single pressing and releasing cycle is shown in Figure 5c. An instant positive peak of ~170 V is generated during the pressing cycle which is quite higher than a negative peak of ~88 V generated in the opposite direction during the releasing cycle. This asymmetric signal generation is caused by the slower release of the device when the force is removed. This indicates the triboelectric output voltage is highly dependent on the contact-releasing speed. Moreover, for comparison, a TENG device of the same size and construction was fabricated, but without b-PEN and with the Al layer as a tribopositive layer; this device generated the maximum open-circuit voltage of ~42 V and a current of ~1.24 µA, as shown in Figure 5a,b. These results indicate that b-PEN is an efficient tribopositive material that can be considered for the fabrication of high-performance TENG devices.

Apart from the voltage and current output, one of the important factors in evaluating the performance of a TENG is its instantaneous power generation capability. The maximum power was calculated by measuring the current of the device at different load resistances (*P* = *I*^2^*R*, where *P* is the power, *I* is the current, and *R* is resistance). The short-circuit current and the instantaneous power of the TENG device are shown in Figure 5d. It can be noted that the current decreases with increasing load resistance. On the contrary, the corresponding power density increases with increasing load resistance up to 10 MΩ and then decreases. The b-PEN/PTFE-based TENG device delivered a maximum instantaneous power density of 72 µW/cm^2^ at 10 MΩ. Hence, the output electrical performance of the device (185 V, 4.52 µA, and 72 µW/cm^2^) confirms that b-PEN is a promising material for energy harvesting. Furthermore, to determine the origin of the generated voltage signals, a phase-change analysis of the b-PEN/PTFE-based TENG device was performed, as shown in Figure 5e. The polarity of the generated voltage signals shifts by 180° when the output connections are altered. This analysis ensures that the signals generated from the TENG device are obtained without any noise from the outside environment. The durability of the TENG device was analyzed using a stability test performed by repeating the contact–separation cycle for 2000 cycles (~6 min). As shown in Figure 5f, the device exhibits negligible changes in its output levels, thus suggesting its outstanding stability and reliability in energy harvesting.

The performance of the b-PEN TENG is compared with other TENG devices based on tribopositive biomaterials as shown in Table 1. The output performance of the proposed b-PEN TENG in a non-processed state is comparable with the previous reports where the materials are processed before being employed as an energy harvester. The triboelectric output performance of the proposed b-PEN TENG device can be further optimized for higher power generation by adapting advanced coating methods such as spray coating to increase the surface roughness and adhesion [56]. Robust structural design and selection of the opposite affinity material with electron-accepting capabilities also play crucial roles in enhancing the power generation performance [28].

**Figure 6:**
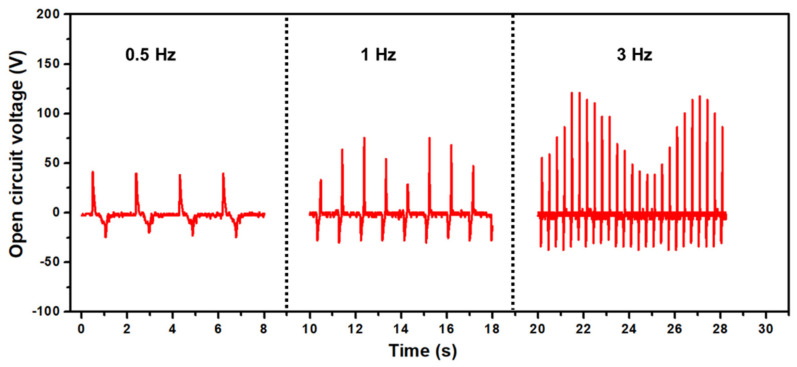
Frequency−dependent open−circuit voltage of b-PEN TENG at 0.5, 1, and 3 Hz conditions.

Frequency-dependent electrical performance is of utmost importance in energy harvesting devices depending on the specific applications and conditions. Specifically, low-frequency operatable TENG devices are crucial for efficiently harvesting the energy from the mechanical vibrations or movements that occur at lower frequencies. Human movements such as walking, hand movements, and other subtle movements usually generate lower frequencies, thus TENGs are suitable for wearable devices operating at lower mechanical deformation rates. The frequency-dependent open-circuit voltage performance is evaluated at the same conditions of force (5 N) with 0.5, 1, and 3 Hz frequencies as shown in Figure 6. As shown at 0.5 Hz, the output voltage generated by b-PEN TENG was ~48 V. As the frequency increased from 1 Hz to 3 Hz, the maximum output voltage increased to ~75 and ~101 V, respectively. The triboelectric output performance is highly dependent on the surface charges and tapping speed of the TENG device. The surface charge accumulation and transfer can be enhanced by increasing the pressing and releasing speed of the top and bottom triboelectric layers. With lower frequencies, the pressing speed decreases, resulting in lower surface charges. As the frequency increases, the TENG output reaches a saturation level and starts decreasing at much higher frequencies [64]. This decrease in the output voltage is because limited time is available for the full separation between the top and bottom triboelectric layers of the TENG device during the fast-pressing cycles, thus reducing the separation distance between the top and bottom layers, leading to degradation of voltage at higher frequencies. The proposed b-PEN TENG device shows quite satisfactory output performance at low frequencies from 0.5 Hz up to 5 Hz which can be suitable for mechanical energy harvesting of human motion.

**Figure 7:**
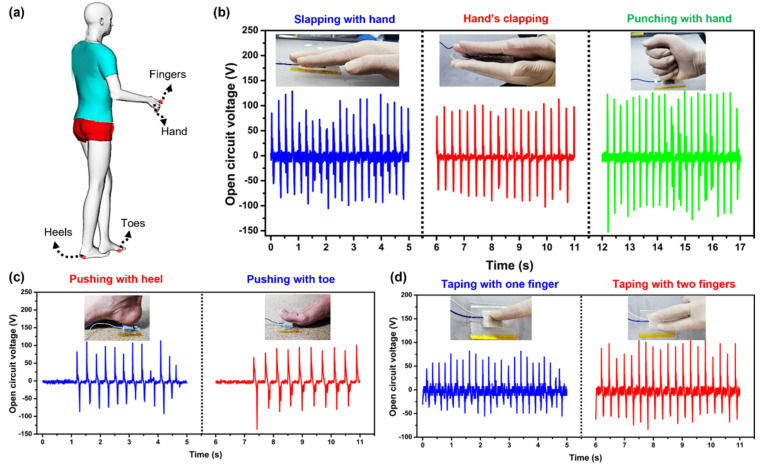
The energy−harvesting capability of the b-PEN TENG device: (**a**) scheme of energy harvesting from mechanical motions of different body parts, (**b**) slapping, clapping, and punching with a hand, (**c**) pushing with a foot (toe, and heel), and (**d**) fingertip tapping (one finger and two fingers).

The proposed b-PEN/PTFE-based TENG device has good flexibility and can generate sufficient voltage, current, and power for use as a wearable power source. The dynamic biomechanical energy-harvesting performance of the fabricated TENG device was determined by actuating it with different parts of the human body, as shown in Figure 7a. The TENG device generated a maximum of ~130, ~125, and ~150 V when slapped with a hand, clapped, and punched, respectively, as shown in Figure 7b. Additionally, the fabricated TENG device was used to harvest energy under the foot, toe, and heel, as illustrated in Figure 7c. Furthermore, the repeated tapping of the TENG device with a single finger at a constant force yields ~70 V, whereas more than ~100 V is generated when the device is tapped with two fingers, as shown in Figure 7d. The harvesting of finger-tapping energy can be applied to touch screens and computer keyboards.

**Figure 8:**
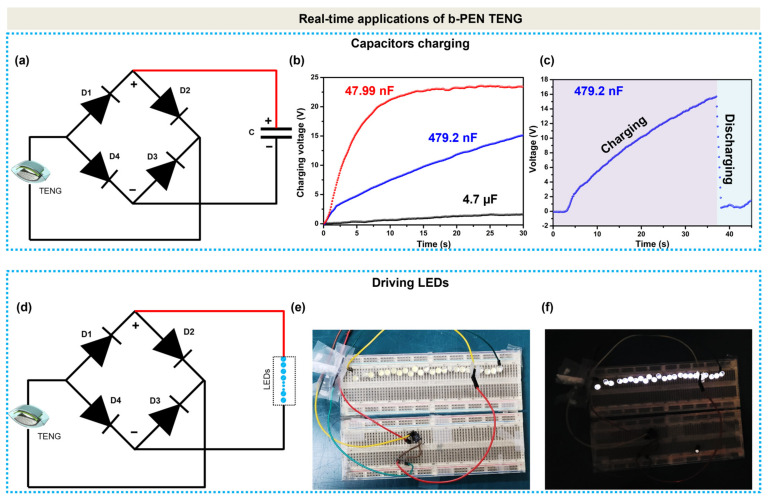
(**a**) Circuit diagram of the b-PEN TENG connected to the rectifier circuits for charging different capacitors, the red line indicates the positive wire connection. (**b**) Charging behavior of 47.99 nF, 479.2 nF, and 4.7 µF capacitors. (**c**) Charging and discharging behavior of the 479.2 nF capacitor. (**d**) Circuit diagram of the b-PEN TENG connected to light-emitting diodes (LEDs) via the rectifier circuit. (**e**,**f**) Photos of LEDs powered by b-PEN TENG in off and on states.

Low-power-operated electronic equipment is often powered by a constant direct-current power source. However, the TENG device generates an alternating current. Therefore, the TENG-generated signals must be rectified using a full wave bridge rectifying circuit, as shown in Figure 8a, to charge the energy-storing device. Figure 8b shows the charging capability of the b-PEN TENG device, applied to different capacitors. The commercially available capacitors with the capacitances of 47.99 nF, 479.2 nF, and 4.7 µF were charged up to the voltages of 22.5, 13, and 2 V, respectively, by repeatedly actuating the TENG device with the pneumatic cylinder for 30 s. Within the same charging time, the 47.99 nF capacitor was the fastest to charge among the utilized capacitors. Similarly, the charge and discharge of the 479.2 nF capacitor with a connected load connected to it are exhibited in Figure 8c. The TENG-powered capacitor is gradually charged up to 15 V for 37 s and quickly discharges when the TENG device stops operating. Hence, the proposed b-PEN TENG demonstrates a good capability to power low-power electronic devices for continuous use. To illustrate the self-powered applications of the b-PEN TENG, 24 commercial LEDs (maximum power dissipation 10 mW and full light illumination current of 20 mA) were connected in series with the b-PEN TENG via a rectifier circuit, as shown in Figure 8d. The TENG device, when actuated using mechanical force, can easily light up 24 LEDs, as shown in Figure 8e,f. The above results indicate that b-PEN (antibiotic) is a promising tribopositive material for triboelectric energy harvesting.

## 4. Conclusions

In conclusion, in this study we have explored the unique potential of benzylpenicillin sodium salt (b-PEN), an antibiotic, as a tribopositive material, showcasing its successful application in triboelectric nanogenerators (TENGs). By employing b-PEN powder in conjunction with polytetrafluoroethylene (PTFE) with opposite electron affinities as triboelectric layers, we have crafted a high-performance TENG device. The electron-donating capability of b-PEN, facilitated by the lone pairs of electrons on its sulfur atom, carbonyl, and amino functional groups, enables the generation of electric potential. The resulting b-PEN TENG device, sized at 2 × 2 cm^2^, exhibits impressive electrical parameters, including an open-circuit voltage of 185 V, a short-circuit current of 4.52 µA, and a maximum power output of 72 µW/cm^2^. Importantly, the fabricated device maintains stable electrical performance over 2000 cycles under a continuously applied force. Furthermore, the b-PEN TENG device demonstrates practical applicability by efficiently charging energy-storing devices, such as capacitors, and powering up to 24 commercial LEDs. We have utilized the abundantly available biomaterial penicillin G sodium salt with no pre-processing before employing it as a tribomaterial, which is suitable for cost-effective energy harvesting devices. The use of antibiotic materials like b-PEN has notable advantages including their electron-donating capability, sustainable and abundant sources, chemical stability, biocompatibility, versatility in applications, and innovative material selection. The demonstrated performance of antibiotic-based TENGs highlights their potential for contributing to the advancement of electronic technologies, paving the way for novel devices with enhanced functionalities.

## Figures and Tables

**Table 1:** Comparison of tribopositive materials of previously reported biomaterials-based TENGs.

Year	Tribopositive Material	Tribonegative Material	Voltage (V)	Power Density (µW/cm^2^)	Ref
2016	Rice husk	PTFE	270	8.4	[57]
2018	Soy protein	PI	~5	1.26	[58]
2020	Sugar	PTFE	95.68	3.33	[59]
2020	Silk cocoon	PTFE	390	0.002	[60]
2021	NaCl powder	PTFE	198	11.202	[61]
2021	Paracetamol	PTFE	561	163	[62]
2022	Starch	PEF	151	113.2	[63]
2023	Penicillin-G Sodium salt	PTFE	185	72	This work

## Data Availability

Data are available upon request.
